# Low contrast visual acuity testing is associated with cognitive performance in multiple sclerosis: a cross-sectional pilot study

**DOI:** 10.1186/1471-2377-13-167

**Published:** 2013-11-08

**Authors:** Laura Wieder, Gunnar Gäde, Luisa M Pech, Hanna Zimmermann, Klaus-Dieter Wernecke, Jan-Markus Dörr, Judith Bellmann-Strobl, Friedemann Paul, Alexander U Brandt

**Affiliations:** 1 NeuroCure Clinical Research Center, Charité – Universitätsmedizin Berlin, Berlin, Germany; 2 SOSTANA GmbH and Prof. em. Institute of Medical Biometrics and Clinical Epidemiology, Charité – Universitätsmedizin Berlin, Berlin, Germany; 3 Clinical and Experimental Multiple Sclerosis Research Center, Charité – Universitätsmedizin Berlin, Berlin, Germany; 4 Department of Neurology, Charité – Universitätsmedizin Berlin, Berlin, Germany

**Keywords:** Multiple sclerosis, Cognition, Vision, Vision tests, Contrast sensitivity, Neuropsychological tests, Optical coherence tomography, Retinal nerve fiber layer

## Abstract

**Background:**

Cognitive impairment and visual deterioration are two key clinical symptoms in MS and affect 50 to 80% of patients. Little is known about the influence of cognitive impairment on visual tests recommended for MS such as low contrast sensitivity testing. Our objective was to investigate whether low contrast sensitivity testing is influenced by cognitive impairment in multiple sclerosis (MS) patients.

**Methods:**

Cross-sectional study including 89 patients with relapsing-remitting MS. All patients received cognitive evaluation using Rao’s Brief Repeatable Battery of Neuropsychological Testing (BRB-N). Visual assessments included low contrast sensitivity (CS) by functional acuity contrast testing and high contrast visual acuity (VA) using ETDRS charts. Retinal morphology as visual impairment correlate was measured using retinal nerve fiber layer (RNFL) thickness by optical coherence tomography.

**Results:**

In combined analyses using generalized estimating equation models, Paced Auditory Serial Addition Test (PASAT) and RNFL as well as and the Symbol Digit Modalities Test (SDMT) and RNFL predicted CS. To further control for a potential influence of the anterior visual system we performed partial correlation analyses between visual function and cognitive function test results but controlling for RNFL. Even when controlling for RNFL, CS was associated with PASAT performance and SDMT performance.

**Conclusion:**

Our data show that: a) cognitive impairment and performance in visual function tests such as low contrast sensitivity testing are associated; b) the main cognitive domains correlating with visual test performance are information processing speed and, to a lesser degree, memory; This preliminary data needs to be substantiated in further studies investigating patients with a higher cognitive burden, healthy controls and in longitudinal settings.

## Background

Multiple sclerosis (MS) is the most prevalent autoimmune disorder of the central nervous system and presents with a variety of neurologic symptoms [1]. About 80% of patients experience visual dysfunction which is the presenting symptom in every second patient [2]. Even in visually asymptomatic patients, subclinical visual system deficits can be detected using high-sensitivity methods like high-pass resolution perimetry [3] or high resolution optical coherence tomography (OCT) [4,5].

Due to the frequency of visual deficits in MS, assessment of visual function plays an important role in clinical trials and clinical practice [6]. Visual symptoms are usually assessed during neurological examination with a combination of history taking, finger-perimetric visual field assessment and visual acuity testing using Snellen charts [7]. However, previous studies have shown that detection and quantification of visual dysfunction with high contrast visual acuity testing is insufficient. Particularly, mild changes are easily overlooked [8]. Hence, several recent studies have investigated higher sensitivity tools based on low contrast sensitivity testing for detecting damage to the visual pathway. Here, the Sloan low contrast visual acuity charts [6,9-11] and the Pelli-Robson contrast sensitivity charts [10,12] have been demonstrated as superior to high contrast visual acuity testing in MS. Both visual outcome measures have also been shown to correlate strongly with retinal nerve fibre layer (RNFL) thickness [13], which is an important OCT marker for assessing the structural integrity of retinal axons [14,15]. Likewise, functional acuity contrast testing (FACT), as an alternative method for assessing low contrast sensitivity, was shown to correlate well with retinal axon damage in MS [16]. Consequently, clinician-researchers have suggested including low contrast visual acuity testing in the Multiple Sclerosis Functional Composite (MSFC), the most widely used quantitative clinical assessment tool in MS [9,17,18].

In order to better understand both the benefits and the limitations of low contrast visual function testing, further studies are needed to define their place in the often complicated array of neurological symptoms in MS patients. Sophisticated visual function tests have become increasingly challenging and demanding for the patient. For example, the identification of letters (e.g. Sloan and Pelli-Robson charts) or the direction of grated lines (FACT) most likely requires higher cognitive functions. We therefore hypothesised that test performance is likely not only influenced by visual impairment (in terms of actual morphological damage to the anterior visual system) but also by higher cognitive and executive functions.

Cognitive impairment (CI) is also a common symptom in MS with a prevalence of about 50% [19] and mild cognitive decline can even begin in early MS [20]. Processing speed, working memory and executive function are the cognitive domains mainly affected [19].

In light of this, our study sought to determine how cognitive impairment influences low contrast sensitivity testing. Here we report cross-sectional data from a large prospective cohort of relapsing-remitting MS patients. To assess morphological visual impairment we determined RNFL thickness [14]. Cognitive function was assessed using Rao’s Brief Repeatable Battery of Neuropsychological Testing (BRB-N) [21].

## Methods

### Patients and study design

For this cross-section pilot study, eighty-nine patients with RRMS according to the 2005 panel criteria [22] were recruited from the outpatient clinic of the Clinical and Experimental Multiple Sclerosis Research Center at the Charité – Universitätsmedizin Berlin. Inclusion criteria were: definite RRMS, Expanded Disability Status Scale (EDSS) between 0.0 and 6.0, stable immune-modulatory therapy for at least 6 months. Exclusion criteria were: acute relapses and/or steroid treatment three months prior to inclusion, any other ocular diseases with known retinal pathology (i.e. glaucoma), refractive error > ±5 dpt. There were no specific inclusion or exclusion criteria in regard to cognitive or psychiatric status. Patients underwent clinical assessment, neuropsychological testing and visual examination within a three-month period. An overview of the cohort’s demographic details is given in Table 1.

**Table 1:** Overview of cohort’s clinical and visual data

**Subjects**	***N***	**RRMS** **89**
Sex	Male, *N* (%)	36 (40)
	Female, *N* (%)	53 (60)
Age (years)	Mean ± SD	42 ± 9
	Min - Max	25 - 62
Time since diagnosis (months)	Mean ± SD	97 ± 67
	Min - Max	2 - 340
EDSS	Median	2.0
	Min - Max	0.0 - 6.0
RNFL Average (μm)	Mean ± SD	84.5 ± 14.3
	Min - Max	33.2 - 117.4
VA (ETDRS Snellen equivalents)	Mean ± SD	1.05 ± 0.36
	Min - Max	0.20 - 1.60
CS (FACT AUC)	Mean ± SD	1.85 ± 0.32
	Min - Max	0.82 - 2.28
Cognitively impaired (BRB-N z < 0.168)	No, *N* (%)	63 (72)
	Yes, *N* (%)	25 (28)
Fatigue severity scale (FSS)	Mean ± SD	5.0 ± 2.5
	Min - Max	0.0 - 9.0
Fatigued (FSS > = 4.0)	No, *N* (%)	35 (39)
	Yes, *N* (%)	54 (61)
Beck’s depression inventory (BDI)	Mean ± SD	8.1 ± 7.9
	Min - Max	0 - 29
Depressed (BDI > = 30; 19-29; 10-18; 0-9)	Minimal, *N* (%)	60 (69)
	Mild, *N* (%)	14 (16)
	Moderate, *N* (%)	13 (15)
	Severe, *N* (%)	0 (0)

*Abbreviations*: *EDSS* expanded disability status scale, *RNFL* retinal nerve fibre layer thickness, *VA* visual acuity, *ETDRS* early treatment diabetic retinopathy study, *CS* contrast sensitivity, *FACT* functional acuity contrast testing, *AUC* area under the log contrast sensitivity function, *BRB-N* brief repeatable battery of neuropsychological tests.

The study was approved by the local ethics committee of the Charité – Universitätsmedizin Berlin and was conducted in accordance with the Declaration of Helsinki in its current version. All patients gave written informed consent.

### Clinical examination and neuropsychological testing

All participants underwent clinical neurological examination, including the EDSS, under the supervision of a board-certified neurologist [7]. Fatigue was assessed using the Fatigue Severity Scale (FSS) [23]. Patient with a mean FSS score > = 4 were classified as fatigued. Depression was assessed using Beck’s Depression inventory (BDI) [24]. Patients’ depression was classified using the following cutoff-values: Minimal: 0 – 9; Mild: 10 – 18; Moderate: 19 – 29; Severe: > = 30. The BDI was not available for two patients. Patients performed all subtests of the German BRB-N version A under supervision of trained examiners and with best available optic correction [21,25]. Tests were performed as previously described in detail [26]. Briefly, the BRB-N consists of the following subtests: 1) Selective Reminding Testing (SRT)[27], which measures verbal learning and memory in terms of immediate recall (SRT-LTS and SRT-CLTR) and delayed recall (SRT-D); 2) 10/36-Spatial Recall Testing [28], which measures the visio-spatial memory in terms of immediate recall (SPART) and delayed recall (SPART-D); 3) Symbol Digit Modalities Testing (SDMT) [29], which tests information processing speed and concentration; 4) Paced Auditory Serial Addition Testing [30], including the three-second version (PASAT3) and the two-second version (PASAT2), which measures information-processing speed and working memory; 5) Word List Generation (WLG) [31], which tests semantic verbal fluency, verbal production and executive function. BRB-N z-scores were calculated as previously described against normative data for German MS patients using the original script kindly provided by Dr. Scherer [25]. Patients were classified as cognitively impaired when z-score < 1.68 [25].

### Visual acuity and contrast sensitivity

Visual acuity (VA) and contrast sensitivity (CS) were analysed using the “Optec 6500 P vision testing system” (Stereo Optical, Chicago, Illinois). A trained operator performed all examinations in a darkened room. VA was assessed in decimal fractions using Early Treatment Diabetic Retinopathy Study (ETDRS) charts. These results were then summarised by translation into Snellen equivalents. CS was measured using Functional Acuity Contrast Testing (FACT) and was performed monocularly for both eyes with test scores as the last correct grating determined under photopic conditions without glare. Photopic conditions were simulated with 85 cd/m^2^ at target image. FACT evaluation was performed by calculating the Area Under the Log Contrast Sensitivity Function (AUC) as previously described in detail [16]. Briefly, the five contrast sensitivity values at five different spatial frequencies for each measurement were transformed into logarithmic expression and a curve combining all values using a polynomial fit function was calculated. The AUC was then calculated as the area between the lowest and highest spatial frequency under this curve.

### Optical coherence tomography

Optical coherence tomography was performed on both eyes of each patient using a spectral domain OCT (SD-OCT) device (Heidelberg Spectralis, Heidelberg Engineering, Heidelberg, Germany) by experienced operators. Retinal nerve fibre layer thickness (RNFL) was measured using standard protocol with three 3.4 mm circular scans. All scans were reviewed for scan quality according to [32]. The scan with the highest quality or, if equal quality was achieved, an arbitrarily selected one, was included in the analysis.

### Statistical analysis

The correlation between visual and cognitive tests was analysed using generalized estimating equations models (GEE) to account for within-patient inter-eye effects. Combined models using both RNFL and PASAT or RNFL and SDMT as multiple independent variables and CS as dependent variable were analysed using GEE. An estimation of the partial correlation between cognitive tests and visual tests controlled for RNFL was calculated using the following approach: First, GEE analyses were performed, with each parameter as dependent variable and RNFL as independent variable in a normalized fashion. The residuals from these models were then fed into a Spearman’s Rho correlation analysis, producing results comparable to those of a regular partial correlation analysis, but which also take into account within-subject inter-eye effects. Local regression analysis (LOESS) was used to visually estimate influence of cognitive impairment on visual function testing [33]. All statistical analyses were performed using SPSS Statistics 20 (SPSS Statistics Version 20, IBM, Armonk, NY, USA). A type I error level of α = 0.05 was taken to indicate statistical significance. All test results should be considered exploratory data analysis as no previous sample size calculation or adjustment for multiple testing was applied.

## Results

### Correlation between RNFL and visual and cognitive performance

An overview of the OCT and visual function test results is given in Table 1. A summary of the BRB-N scores is given in Table 2. 28% of the patients were cognitively impaired according to the BRB-N. We tested the possible correlation between, firstly, RNFL thickness and visual test results and, secondly, RNFL thickness and cognitive test results using GEE models. As expected, RNFL thickness predicted high contrast VA (B = 0.011, SE = 0.002, p < 0.001) and CS (B = 0.011, SE = 0.002, p < 0.001). In contrast, there was no correlation between RNFL thickness and any of the BRB-N tests (p ≥ 0.223).

**Table 2:** Data from cognitive testing and correlation with visual test performance

					**GEE vs. CS**			**Part. Corr. vs. VA**		**Part. Corr. vs. CS**	
	**Mean**	**SD**	**Min**	**Max**	**B**	**SE**	***P***	**Coefficient**	***P***	**Coefficient**	***P***
SRT-LTS	60	11	24	72	0.008	0.003	**0.007**	0.079	0.333	0.154	0.093
SRT-CLTR	55	15	4	72	0.004	0.003	0.103	0.081	0.319	0.160	0.080
SRT-D	11	2	2	12	0.057	0.021	**0.008**	0.188	**0.021**	0.336	**<0.001**
SPART	23	5	10	30	0.019	0.007	**0.012**	0.240	**0.003**	0.215	**0.018**
SPART-D	9	2	3	10	0.025	0.025	0.321	0.154	0.058	0.055	0.548
SDMT	56	12	32	85	0.010	0.002	**<0.001**	0.338	**<0.001**	0.343	**<0.001**
PASAT3	50	9	24	60	0.014	0.003	**<0.001**	0.171	**0.035**	0.286	**<0.001**
PASAT2	40	9	19	59	0.009	0.004	**0.013**	0.157	0.082	0.240	**0.019**
WLG	28	7	14	42	0.014	0.005	**0.008**	−0.056	0.496	0.221	**0.016**
BRB-N z	0.685	1.270	−4.084	2.715	0.083	0.029	**0.004**	0.142	0.083	0.249	**0.006**

*Abbreviations*: *SRT-LTS* selective reminding test long term storage, *SRT-CLTR* selective reminding test consistant long term retrieval, S*RT-D* selective reminding test delayed recall, *SPART* spatial recall test, *SPART-D* spatial recall test delayed recall, *PASAT* paced auditory serial addition test, *WLG* word list generation, *BRB-N* brief repeatable battery of neuropsychological tests, *VA* high contrast visual acuity, *CS* low contrast sensitivity.

Significant P values are printed in bold.

Subsequently, we investigated whether visual function testing correlated with cognitive test performance. Here, CS correlated significantly with BRB-N z-scores and most subtests, except SRT-CLTR and SPART-D (Table 2).

### Correlation between contrast sensitivity and cognition when controlling for RNFL

**Figure 1:**
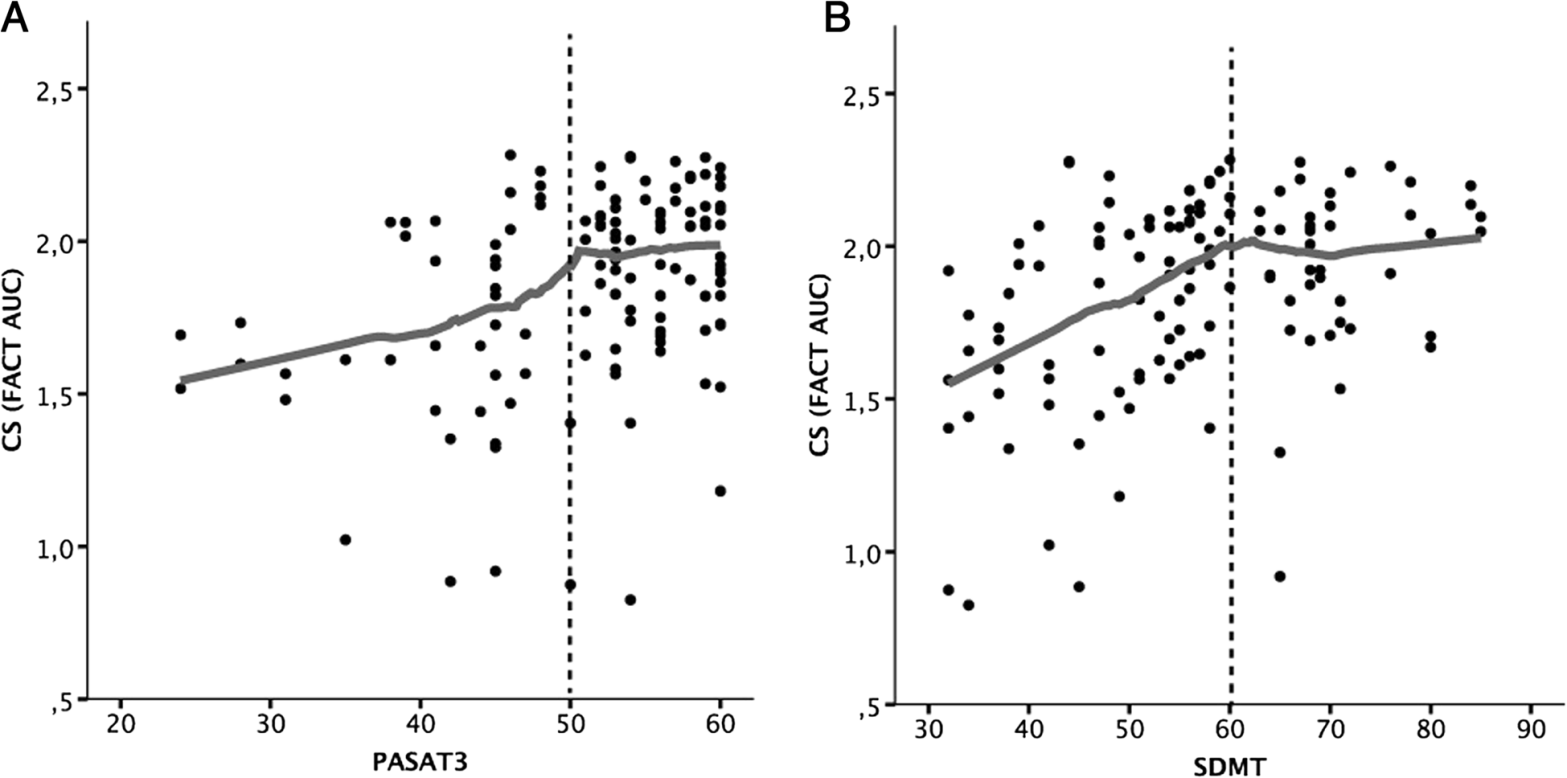
**Local regression analysis of cognitive influence on low contrast sensitivity testing. A)** Correlation between PASAT and CS, **B)** Correlation between SDMT and CS. Grey lines are from LOESS analysis. Horizontal LOESS lines represent areas with low or no correlation; rising LOESS lines represent areas with correlation between cognitive function and visual function test performance. For better visibility dotted lines separate the areas with an influence (left sides) and without influence (right sides). Data from partial correlations controlling for retinal nerve fibre layer thickness show a similar result (not shown). Abbreviations: LOESS, local regression analysis; CS, low contrast sensitivity; PASAT, paced auditory serial addition test three second version; SDMT, symbol digit modalities test; FACT, functional acuity contrast testing; AUC, area under the log contrast sensitivity function.

To determine whether the anterior visual system (i.e. retinal damage) influences the correlations discussed above, we performed partial correlation analyses between visual function and cognitive function tests but controlling for RNFL. This way, an influence of actual retinal damage to the performance on visual tests should be eliminated. When controlling for RNFL, a clear correlation was determined between VA and SRT-D, SPART, SDMT and PASAT3. Likewise, CS correlated with the latter cognitive tests, as well as with PASAT2 and WLG (Table 2). In the involved domains, coefficients were moderate and roughly twice as high for cognitive influence on CS as for cognitive influence on VA with the exception of SDMT, a visually dependent cognitive test. SDMT correlation with visual function testing was highest among all tests with r = 0.34 both in CS and VA testing (Figure 1).

### Cognitive function and RNFL together predict contrast sensitivity

To determine whether retinal morphology and cognitive function have an additive effect, we assessed the combined effect of cognitive test performance and RNFL on visual function testing. Detailed results are given in Table 3. In summary, in a multiple GEE both PASAT and RNFL predicted CS. Similarly, both SDMT and RNFL correlated with CS. BRB-N and all subtests except SRT-CLTR, SPAT-D and WLG were also significantly predictive of CS.

**Table 3:** Results from combined GEE predicting low contrast sensitivity test performance

	**Cognitive test**			**RNFL average**		
	**B**	**SE**	***P***	**B**	**SE**	***P***
SRT-LTS	0.006	0.0028	**0.024**	0.011	0.0023	**<0.001**
SRT-CLTR	0.004	0.0022	0.064	0.011	0.0023	**<0.001**
SRT-D	0.047	0.0179	**0.009**	0.011	0.0024	**<0.001**
SPART	0.019	0.0077	**0.015**	0.011	0.0023	**<0.001**
SPART-D	0.026	0.0226	0.244	0.012	0.0024	**<0.001**
SDMT	0.007	0.0021	**<0.001**	0.001	0.0023	**<0.001**
PASAT3	0.012	0.0038	**0.002**	0.011	0.0025	**<0.001**
PASAT2	0.008	0.0039	**0.035**	0.001	0.0031	**<0.001**
WLG	0.009	0.0047	0.052	0.011	0.0024	**<0.001**
BRB-N z	0.003	0.0011	**0.017**	0.011	0.0023	**<0.001**

*Abbreviations*: *SRT-LTS* selective reminding test long term storage, *SRT-CLTR* selective reminding test consistant long term retrieval, *SRT-D* selective reminding test delayed recall, *SPART* spatial recall test, *SPART-D* spatial recall test delayed recall, *PASAT* paced auditory serial addition test, *WLG* word list generation, *BRB-N* brief repeatable battery of neuropsychological tests, *RNFL* retinal nerve fiber layer thickness.

Significant P values are printed in bold.

## Discussion

Our cross-sectional pilot study investigated the influence of cognitive impairment on visual function testing in MS patients. Our data show that a) cognitive impairment correlates with performance in visual function tests such as low contrast sensitivity testing; b) the main cognitive domains correlating with visual test performance are information processing speed and, to a lesser degree, memory.

When quantifying clinical symptoms in MS patients, the examiner has to carefully consider bias from other functional domains. For example, in cognitive testing, motor impairment can impede the provision of a signal by pressing a button, while visual impairment can hamper the recognition of figures or shapes. Overall, little attention has been paid to the interplay between cognitive and visual function and its potential influence on clinical testing. Two previous investigations studying the converse of our study focus were able to demonstrate the validity of their hypothesis, namely, that visual acuity indeed influences performance in visually dependent cognitive tests like SDMT [34,35]. The authors’ conclusion was that visual dysfunction potentially impairs test performance in visually dependent cognitive tests. Changes in cognitive function might therefore be over- or underestimated when switching from non-visually dependent tests like PASAT to visually dependent tests like SDMT. Consequently, visual pre-screening is currently recommended when applying and interpreting visually dependent cognitive test results [36].

But can on the other hand cognitive impairment influence visual function test results? A recent study investigating auditory-based cognitive tests (i.e. PASAT) in combination with low contrast visual acuity showed that the latter correlates strongly with visually dependent but also non-visually dependent cognitive test results [37]. However, crucially, the authors did not compare their findings to actual damage to the visual pathway [13]. By not factoring in morphological assessment such as by RFNL thickness, the correlation between cognitive impairment and visual function could simply have been a correlate of overall disease progression with visual and cognitive performance declining in parallel, yet independently. This reservation is underlined by the results of a study by Toledo and colleagues, which has shown strong correlation between RNFL thickness and cognitive impairment [38]. Our assessment of the relationship between cognitive and visual test performance excised the influence of optical morphological damage by including RNFL thickness measurements in our analysis. As the correlation between non-visually dependent cognitive test results and visual function tests remained intact, our results provide evidence to support the hypothesis that cognitive impairment influences visual test scores. Particularly our investigation of the correlation between SDMT and VA indicates that more demanding visual tests are affected by cognitive impairment.

The main cognitive domain correlating with visual test performance was speed of information processing, which is a key domain keenly affected in MS [19]. Reduced speed of information processing has been since long recognized as one of the hallmark cognitive domains impaired in MS and is the basis for PASAT and SDMT being used as screening tests for cognitive impairment in MS, since both tests mainly assess this domain [36]. Studies have suggested that reduced processing speed might be the result of alterations in participating neural networks due to damaged white and grey matter [39]. Put simply, compared to health controls, MS patients take more time to complete tasks in cognitive testing, because they require greater neural recruitment [40]. Our results might be explained by the influence of similar neural network disruptions in cognitive domains when processing visual tests, suggesting that investigation of neural recruitment also in higher cognitive domains during low contrast visual acuity testing may well prove a valuable avenue of further investigation.

Other studies have shown that changes in the attention network might also contribute to this reduction in processing speed [41]. For example, Motoyoshi showed in a recent study that diminishment of attention by inclusion of a concurrent task reduces CS for grating with low temporal frequencies, and concluded that CS might well be generally modulated by attention [42]. However, this finding could be specific to the grating charts used in our and also Motoyoshi’s study and might not apply to letter-based low contrast acuity testing like that of the Sloan test.

Caveats of our study include the lack of a healthy control group. As a result, our study cannot determine whether the observed influence is disease specific (as the focus on processing speed suggests) or performance specific (meaning that also healthy individuals with low cognitive performance might show similar associations). Additionally, our study included mostly patients with mild or no cognitive impairment. A study investigating patients with an on average higher cognitive and clinical burden would allow more meaningful assessment of the dimension of cognitive influence on visual test results within a range of higher cognitive impairment. In this regard it would be of interest, how our results relate to general disease activity markers i.e. from magnetic resonance imaging. Unfortunately these were not available for this cohort, which is another weakness of this study. As a result, this study cannot answer the question, how much of the reported effects might be based on overall disease progression (i.e. both the visual and cognitive systems progress in parallel but independently of each other).

Furthermore, RNFL only represents a limited portion of the visual pathway and results might not include changes in areas such as the posterior visual pathway [43] between the lateral geniculate corpora and the occipital cortex, caused not by optic neuritis (ON) [44], but by incidental lesions [45]. Thus, our correction for RNFL may not have included the entirety of morphological damage to the visual system in our study participants. This could be addressed in a subsequent study by employing e.g. visually evoked potentials (VEP) to accurately define the influence of the visual pathway on visual test performance using an alternative method [46].

Of note, some discrepancies exist between our study and the study by Toledo et al. [38]. Although sample size, disease severity and applied methods were comparable, Toledo and colleagues found a moderate correlation between RNFL and cognitive impairment in several cognitive domains, whereas our data did not show this correlation. This is most likely explained by the heterogeneity of the disease but should serve as a reminder that our results should be confirmed in a different cohort.

Our findings are relevant both for clinical trials and clinical practitioners. Visual and cognitive impairment are key determinants of disease burden in MS, and ascertaining the impairment of both is important in the assessing of any new immunomodulatory or symptomatic therapy [47]. Although the correlation between cognitive and visual function in our study was only moderate (less than 6% of CS test variance could be explained by cognitive dysfunction based on BRB-N, 12% based on SDMT), the effect might prove highly relevant in longitudinal observations, when changes in cognitive function might manifest as changes in visual test performances. For example, a reduction in cognitive performance could present as a change in visual function. Vice versa, an improvement in cognitive function after an acute relapse could also translate into improved visual function test results [48]. When applying low contrast sensitivity testing as outcome measure in clinical trials [49], careful investigation of possible confounders from cognitive impairment is therefore warranted. Of note, the shown correlation between cognitive performance and visual test results does not prove a causal relationship. This cross-sectional pilot data has therefore only a limited explanatory power. A longitudinal study is therefore currently underway.

## Conclusion

In summary, our study shows that visual and cognitive function are closely linked in MS. Specifically, cognitive impairment might bias the results of challenging tasks designed to assess visual acuity, such as those based on low contrast visual acuity testing. This correlation is not only a result of overall disease progression, but is strongly dependent on both visual and cognitive function *per se* since the effect persisted after correcting for actual morphological damage to the visual system.

## Competing interests

The authors declare that they have no competing interests.

## Authors’ contributions

LW performed and analyzed cognitive assessments, participated in the statistical analysis and wrote the manuscript. GG and LMP performed cognitive assessments and visual function tests. HZ performed OCT and visual function tests. KDW planned the statistical analysis. JMD and JBS were responsible for patient recruitment and clinical assessment. FP planned the study and revised the manuscript. AUB planned the study, planned and performed the statistical analysis and wrote the manuscript. All authors provided important intellectual content during the study and revising the manuscript. All authors read and approved the final manuscript.

## Pre-publication history

The pre-publication history for this paper can be accessed here:

http://www.biomedcentral.com/1471-2377/13/167/prepub
